# Insight into novel anti-mucormycosis therapies: investigation of new anti-mucormycosis laser-induced photodynamic therapy based on a sulphone bis-compound loaded silica nanoemulsion

**DOI:** 10.1039/d3ra02775a

**Published:** 2023-07-10

**Authors:** Mohamed Abdelraof, Mohamed Fikry, Amr H. Hashem, Mehrez E. El-Naggar, Huda R. M. Rashdan

**Affiliations:** a Microbial Chemistry Department, Biotechnology Research Institute, National Research Centre Dokki Cairo 12622 Egypt; b Ultrafast Picosecond Laser Lab, Physics Department, Faculty of Science, Cairo University Giza 12613 Egypt mfikry@sci.cu.edu.eg; c Egypt Nanotechnology Center (EGNC), Faculty of Nanotechnology for Postgraduate Studies, Cairo University El-Sheikh Zayed 12588 Egypt; d Botany and Microbiology Department, Faculty of Science, Al-Azhar University Cairo 11884 Egypt; e Institute of Textile Research and Technology, National Research Centre 33 El Bohouth St, Dokki Giza 12622 Egypt; f Chemistry of Natural and Microbial Products Department, Pharmaceutical and Drug Industries Research Institute, National Research Centre 33 El Buhouth St, Dokki 12622 Giza Egypt hudadawoud20@yahoo.com

## Abstract

For drug delivery applications, silica nanoemulsion encapsulated with organic compounds are becoming increasingly more desirable. Therefore, the emphasis of this research was on the synthesis of a new potent antifungal drug-like candidate (1,1′-((sulfonylbis(4,1-phenylene)bis(5-methyl-1*H*-1,2,3-triazole-1,4-diyl))bis(3-(dimethylamino)prop-2-en-1-one), SBDMP), the chemical structure of which was confirmed on the basis of its spectral and microanalytical data. Then, silica nanoemulsion loaded with SBDMP was prepared using Pluronic F-68 as a potent surfactant. The particle shape, hydrodynamic size, and zeta potential of the produced silica nanoemulsion (with and without drug loading) were assessed. The antitumoral activity of the synthesized molecules showed the superiority of SBDMP and silica nanoemulsion with and without SBDMP loading against *Rhizopus microsporous* and *Syncephalastrum racemosum*. Subsequently, the laser-induced photodynamic inactivation (LIPDI) of Mucorales strains was determined using the tested samples. The optical properties of the samples were investigated using UV-vis optical absorption and the photoluminescence. The photosensitivity of the selected samples appeared to enhance the eradication of the tested pathogenic strains when exposed to a red (640 nm) laser light. The optical property results verified that the SBDMP-loaded silica nanoemulsion has a high depth of penetration into biological tissues due to a two-absorption photon (TAP) mechanism. Interestingly, the photosensitizing of the nanoemulsion loaded with a newly synthesized drug-like candidate, SBDMP, opens up a new route to apply new organic compounds as photosensitizers under laser-induced photodynamic therapy (LIPDT).

## Abstract 2

For drug delivery applications, silica nanoemulsion encapsulated with organic compounds are becoming increasingly more desirable.

## Graphical abstract

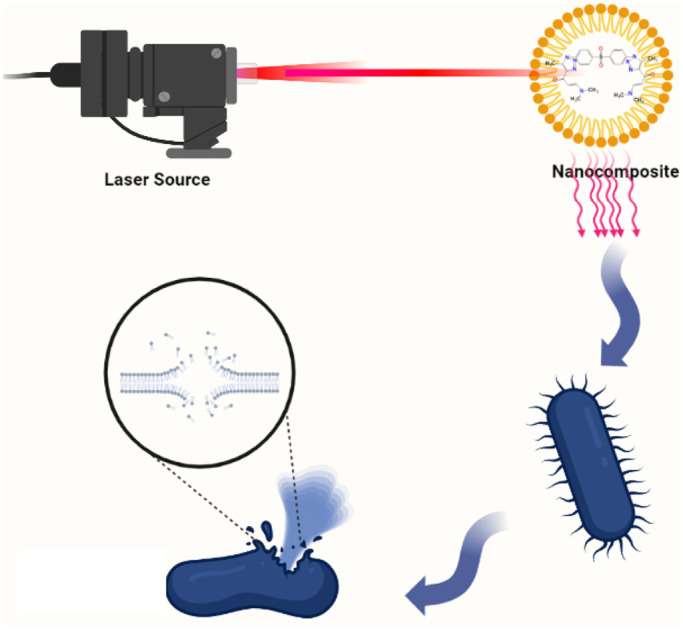

## 1. Introduction

The rapid development of fungal resistance toward classical antibiotics has triggered mortality among immunocompromised patients. Among the extremely virulent fungal pathogens, rare opportunistic Mucorales strains naturally exist in soils.^1^*Rhizopus*, *Mucor*, and *Syncephalastrum* are the most widespread genus that are associated with mucormycosis disease. Mucormycosis is one of the most concerning diseases due to its rapid spread and has several mechanisms to resist classical antifungal antibiotics, particularly among immunocompromised patients.^2,3^ Rapid genetic modification of the Mucorales strains is considered to be the main resistance mechanism of common antifungal agents such as fluconazole. In addition, the rapid proliferation of these pathogens is the most common feature of their invasiveness in the infected tissues in patients with altered immunity (*i.e.* immunocompromised).^4^ Unfortunately, the traditional treatment of mucormycosis has become insufficient against some Mucorales strains, which could be attributed to their having many resistance mechanisms.^5^ Currently, amphotericin B is a major antifungal antibiotic treatment for mucormycosis infection; however, resistance to this agent has been found to be increasing. Furthermore, the side effects of this drug play an important role in restricting its utilization, as it commonly causes nephrotoxicity in many patients.^1^ Therefore, using classical antifungal agents against Mucorales species is frequently sub-optimal and not suitable for some pathogenic strains.^6^ In this respect, new advanced strategies must be designed to achieve the rapid complete eradication of Mucorales pathogens *via* preventing their rapid proliferation. Laser-induced photodynamic therapy (LIPDT) is one of the most efficient techniques that is extensively applied to eradicate multi-drug resistant microbial pathogens in the presence of photosensitizer (PS) compounds.^7^ Applying LIPDT against microbial pathogens is based on the use of PS molecules that absorb laser light of various wavelengths, which induces them in an excited state and produces a series of reactive oxygen species (ROS).^8^ In comparison with traditional antifungal antibiotic agents, LIPDT has the advantage of selectivity, as the irradiation can be spatially directed into the fungal cell and allow the excited PS to penetrate inside it.^9^

Although most of the photosensitizer compounds are dyes, which exhibit a significant response toward irradiation by laser light and generate a huge amount of ROS, they are mostly restricted in treatments, especially those that need to reach the bloodstream owing to their cytotoxicity even at low concentrations. For this purpose, discovering a novel photosensitive drug characterized as edible and biocompatible that can be loaded into a drug delivery system is a new idea not studied previously. Interestingly, drug delivery strategies based on nanotechnology have attracted great consideration from formulation and drug delivery scientists owing to the significant characteristics of nanocarriers, with the aim of limiting their side effects, improving their biocompatibility, and enhancing the therapeutic efficacy and safety of encapsulated drugs.^10^ Additionally, the utilization of nanocarriers in the delivery of targeted drugs improves the photosensitization of the drugs and increases their accumulation at infected sites *via* enhanced permeability and retention effects.^9^ As known, nanoparticles can be used for controlling the release of drugs. Nanoparticles have superior features such as high surface area, orderly structure, and huge pore volume. Due to these properties, silica nanoparticles have been used extensively in the domains of drug delivery throughout the past few decades. Owing to their biocompatibility and biodegradability, silica nanoemulsion are preferred as carriers for the efficient loading and delivery of pharmaceutical compounds that have hydrophobic features. Moreover, dapsone, chemically known as 4,4′-diaminodiphenylsulfone, is an old commercially-available widely-used broad-spectrum synthetic sulfone-based antibiotic.^11–19^ Dapsone exhibits potent antimicrobial,^20^ anti-inflammatory,^21^ and antiprotozoal efficacy and is widely employed for the treatment of different skin disorders, such as leprosy and acne,^22^ as well as the skin issues that arise in gonorrhea.^18^ In addition, dapsone is mainly utilized in the preparation of topical treatments for dermatitis herpetiformis and acne vulgaris.

The main specifications of laser light (monochromatic, coherent, and lower beam divergence), and adjustability of laser parameters (wavelength, output power, irradiance, mode, and illumination time) makes it an ideal light source to use in many applications rather than filtered light lamb and light-emitting diodes (LEDs).^23^ These advantages imply that laser wavelength interacts with a photosensitizer in a small area and avoids overheating bodily tissue. Using optical fibers, the laser light can be easily transported over long distances into internal tumors inside body cavities or through the lumen of needles into the illuminated tissue.^7,24,25^ The thermal interaction of lasers is considered to be one of the significant requirements of the laser light employed in LIPDT.^26^

This study was designed to synthesize a new dapsone-based drug-like candidate (SBDMP). Then, a simple one-pot silica nanoemulsion was prepared utilizing an effective surfactant with the aid of ultrasonication process. The as-prepared SBDMP-loaded silica nanoemulsion was prepared in nanoform with good disparity as a novel composite. Small-sized spherical and well stabilized silica nanoemulsion with and without drug (SBDMP) loading were prepared for the first time. Therefore, a new anti-mucormycosis strategy using the LIPDT approach using SBDMP-loaded silica nanoemulsion was designed against two models of Mucorales strains, *Rhizopus microsporous* and *Syncephalastrum racemosum*. Investigation of the response of SBDMP-loaded silica nanoemulsion toward different irradiation wavelengths, powers, and determination of intracellular ROS at different times was also carried out for the first time.

## 2. Materials and methods

### 2.1. Chemistry

#### 2.1.1. Raw materials

Dapsone, sodium azide, petroleum ether 40–60 °C, absolute methanol, *N*,*N*-dimethylformamide dimethyl acetal (DMF-DMA) and dry toluene were procured from Sigma-Aldrich. Tetraethyl orthosilicate (TEOS) and Pluronic F-68 were purchased from Loba Chemie Pvt, Ltd, Mumbai, India. All chemicals and solvents were used without further purification.

#### 2.1.2. Characterization instruments

All melting points were uncorrected and measured using an electrothermal device.

The infrared (IR) spectra were recorded (KBr discs) using a Shimadzu FT-IR 8201 PC spectrophotometer. ^1^H and ^13^C nuclear magnetic resonance (NMR) spectra were recorded in (CD_3_)_2_SO solutions on a Bruker 500 FT-NMR system spectrometer, and the chemical shifts are expressed in ppm units against tetramethylsilane (TMS) as an internal reference. Mass spectra were recorded on a GC-MS QP1000 EX Shimadzu mass spectrometer. Elemental analyses were carried out at the Microanalytical Center of Cairo University.

The as-prepared nanoemulsions loaded with and without SBDMP were assessed in terms of particle shape using transmission electron microscopy (TEM, JEOL Ltd., Tokyo, Japan). The samples were characterized at different magnifications. In order to prepare TEM samples, a drop of the nanoemulsion was placed onto a copper-coated grid and left in air for drying before evaluation.

The average diameter and zeta potential of the prepared nanoemulsion samples were measured using a particle size analyzer (Nano-ZS, Malvern Instruments Ltd., UK). Before assessment, the nanoemulsion samples were sonicated for 20 min.

The optical absorbance of the samples suspended in water was measured using a V-630 JASCO UV-vis-NIR spectrophotometer over a wavelength range from 190 to 1100 nm at a scan rate of 1000 nm min^−1^ and a cell length of 10 mm.

A highly = sensitive Shimadzu Europe – RF-5301 spectrofluorophotometer with a measuring wavelength range from 220 to 900 nm at a scan rate of 5500 nm min^−1^ and cell length of 10 mm was used to measure the photoluminescence (PL) of samples suspended in water.

#### 2.1.3. Synthesis procedures of the target molecule 1,1′-((sulfonylbis(4,1-phenylene)) bis(5-methyl-1*H*-1,2,3-triazole-1,4-diyl))bis(3-(dimethylamino)prop-2-en-1-one) (SBDMP) (2)

A mixture of 1,1′-((sulfonylbis(4,1-phenylene))bis(5-methyl-1*H*-1,2,3-triazole-1,4-diyl))bis(ethan-1-one) (1) (2.3 g, 5 mmol) and *N*,*N*-dimethylformamide dimethyl acetal (DMF-DMA) were heated under reflux in 20 mL of dry toluene for 4–5 h and the reaction was monitored by thin-layer chromatography (TLC). The hot solution was then evaporated to half its volume and cooled. The resulting solid was collected, washed with petroleum ether at 40–60 °C, and recrystallized from acetic acid to give yellow crystals. Yield: 93%, m.p.: 242–244 °C, FT-IR (KBr, cm^−1^): *v* 2876, 2915 (CH), 1688(C=O), 1601(C=C); ^1^H NMR (DMSO-d_6_): *δ* 2.55 (s, 6H, 2CH_3_), 2.85 (s, 6H, 2CH_3_), 3.11 (s, 6H, 2CH_3_), 7.69–7.91 (m, 10H, ArH, CH=CH), 8.26 (d, *J* = 10 Hz, 2H, CH=CH) ppm; ^13^C NMR (100 MHz, DMSO-d_6_): *δ* 9.75 (2CH_3_), 44.50 (4CH_3_), 99.37 (2CH=), 126.53 (2Ar), 129.24 (4Ar), 136.21 (4Ar), 139.65 (2Ar), 141.09 (2Ar), 144.22 (2Ar), 153.29 (2N–CH=), 180.42 (2C=O); MS *m*/*z* (%): 574 (M^+^, 17). Anal. calcd for C_28_H_30_N_8_O_4_S (574): C, 58.52; H, 5.26; N, 19.50 found: C, 58.56; H, 5.22; N, 19.46%.

#### 2.1.4. Preparation of SBDMP-loaded silica nanoemulsion

Silica nanoemulsion with and without SBDMP loading were prepared in the presence of Pluronic F-68 and TEOS as a surfactant and silica precursor, respectively. Firstly, SBDMP (250 mg) was dissolved in 5 mL of dimethyl sulfoxide (DMSO) at 40 °C for 5 min under magnetic stirring. For the preparation of nanoemulsion, the first step was performed by dissolving 2 mL of TEOS in 28 mL of deionized water. Meanwhile, a second step (loaded with SBDMP) was formed by adding the whole volume of the dissolved SBDMP to 15 mL of deionized water containing Pluronic F-68 (2 mL) under vigorous stirring at room temperature. The SBDMP-loaded solution was transferred to an open vial and left overnight to ensure the complete removal of the residual DMSO solvent. Finally, the nanoemulsion was formed by the dropwise addition of the SBDMP-loaded Pluronic F-68 solution to the TEOS solution under vigorous stirring. At the end of the addition, white solution was formed. For the preparation of a nanoemulsion without SBDMP, all the above steps were repeated without the addition of SBDMP and DMSO. The as-prepared nanoemulsion samples were kept in a refrigerator for characterization and utilization.

### 2.2. Anti-mucormycosis activity of the tested molecules

The anti-mucormycosis activity of the synthesized molecules was investigated against the standard Mucorales fungal strains *Rhizopus microsporous* (Accession no. MK623262), and *Syncephalastrum racemosum* (Accession no. MK621186), which were kindly donated by the Mycology culture collection, Plant and Microbiology Dept., Faculty of Science, Al-Azhar University. The Mucorales fungal strains were preliminarily activated using potato dextrose broth (PDB) culture medium at 28 °C for 72 h under shaking conditions, then 50 μL was inoculated into PDA after standard serial dilution and determined the colony-forming units (CFU) per ml of the tested fungi.^2^ Accordingly, after we determined the inoculum concentration, we adjusted it to 10^6^ mL^−1^ to make it constant. The sensitivity of *Rhizopus microsporous* and *Syncephalastrum racemosum* toward different standard antifungal agents, such as fluconazole and amphotericin B, was determined to evaluate the resistance behavior of the tested fungal strains. Subsequently, an agar well diffusion assay was utilized to determine the initial activity of each compound compared with the antifungal agent at 20 μg mL^−1^. The ability of each targeted compound to prevent fungal proliferation was assessed based on the inhibition zone diameter (mm).^27,28^

### 2.3. Laser-induced photodynamic therapy (LIPDT) setup

**Fig. 1:**
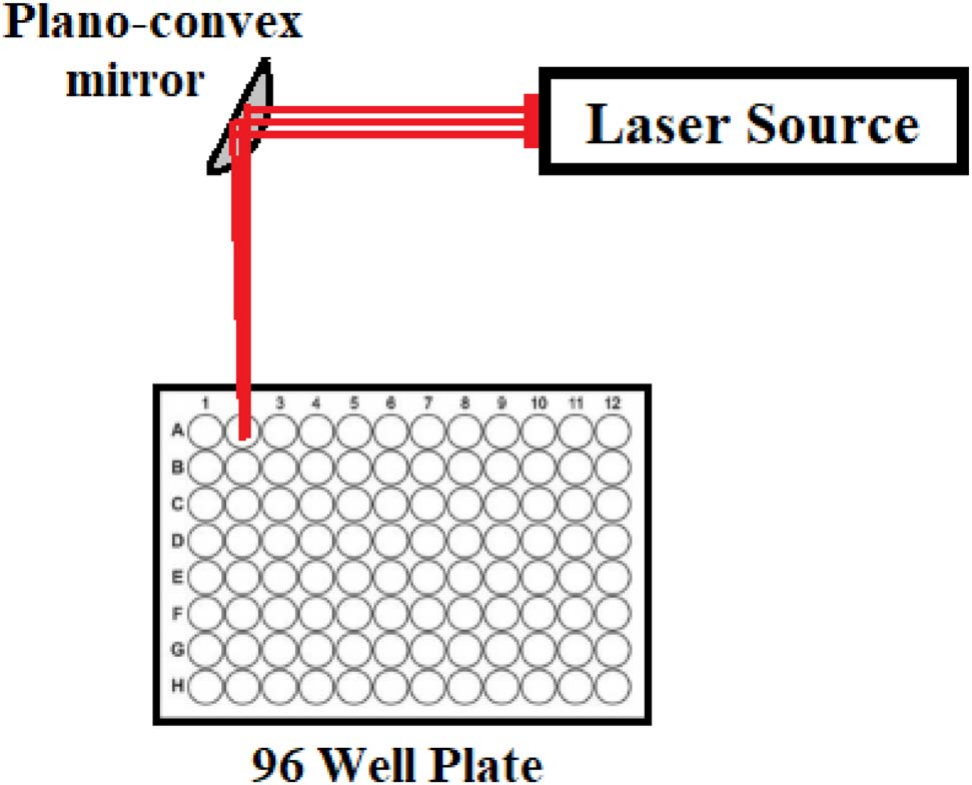
A setup of the laser-induced photodynamic therapy (LIPDT) system.

An ultra-compact CW multipower (1–700 mW) red diode laser module operated at 640 ± 5 nm (model PGL-V-H-640/1 ∼ 700 mW-DC31435, Changchun New Industries Optoelectronics Technology Co., Ltd., Changchun, P. R. China) was used to investigate the photodynamic therapy of SBDMP and SBDMP-loaded silica nanoemulsion on *Syncephalastrum racemosum* and *Rhizopus microsporous* fungi. The laser beam has a diameter of 24 mm and a divergence of 4 mrad. A plano-convex quartz mirror with a focal length of 10 cm was used to focus the beam on the sample on one wall of a 96-wall polypropylene plate at an angle of 45° from the laser axis center,^7,24,29^ with Fig. 1 illustrating the LIPDT system setup.

### 2.4. Laser-induced photodynamic inactivation (LIPDI) studies

The most potent compounds were evaluated as photo-induced molecules in order to determine the eradication efficiency of Mucorales strains. Stock solutions were prepared by dissolving the powder of each compound in DMSO. After filtration through a sterile 0.22 μm membrane, these solutions were stored in the dark for no more than a week before use. Therefore, the selected molecules were tested for their photoactivation potentiality to suppress fungal growth under some laser parameters, such as different laser wavelengths (blue region at 515 nm and red region at 645 nm), different laser power (50 mW and 184 mW), and exposure time (10, 20, and 30 min). Irradiation was performed under aseptic conditions in a laminar flow hood in the dark. After that, the irradiated samples were subjected to serial dilutions, and 100 μL aliquots of each sample were spread into PDA plates and incubated for 48 h at 28 °C. Measurement of the synergistic efficacy between the selected molecules and irradiated conditions was based on the growth inhibition (%) compared to the control (without any treatment) through the CFU procedure.^2^ The evaluation of CFU was carried out according to the following equation:CFU per mL = fungal colony count in the plate × dilution factor/amount transferred to the plate (mL)

Subsequently, the decrease in fungal growth was determined as a percentage value as follows:Percentage reduction = [(fungal count in the control group CFU per mL) − (fungal count in the application group CFU per mL)] × 100/(fungal count in the control group CFU per mL).^7^

The experiment was divided into three groups, testing the targeted molecules alone, exposed to laser light only against the tested Mucorales strain, and the fungal suspension in the presence of selected compounds under irradiation for 25 min. The PDB mixed with DMSO served as the control growth samples. All experimental conditions were performed in triplicate.

### 2.5. Determination of MIC values of the selected molecules

After the determination of the optimal conditions for the photo-killing activity, the minimum inhibition concentration (MIC) of each molecule was examined in comparison with the standard drug *via* a broth microdilution method.^28^ For this purpose, the known weight of each molecule was dissolved in DMSO to prepare the stock solution. Serial dilutions were carried out and 100 μL aliquots were sequentially dispensed into the microdilution plates to obtain the desired concentrations in the range of 5–400 μg mL^−1^. Each concentration of the tested molecule was investigated under the optimized laser conditions and at the end of the cultivation period 50 μL of the treated sample was diluted and dispersed above the PDA plate and incubated at 28 °C immediately in order to calculate the inhibition percentage by CFU count in comparison to the untreated sample. Determination of the MIC value for each photo-treated compound was identified by the lowest concentration of each compound that yields a reduced number of CFU compared to the untreated samples.^30^

### 2.6. Measurement of intracellular ROS

ROS generation inside the fungal cells as a result of the irradiation activity of the potent molecules with the efficient concentration (*i.e.* MIC) was evaluated using fluorescent 2′,7′-dichlorodihydrofluorescein diacetate (DCFH-DA). Intracellular ROS quantification for each fungal suspension was implemented after being treated under the optimized irradiated conditions. Hydrogen peroxide (H_2_O_2_, 155 μM), a standard peroxide commonly used in a variety of oxidation processes, was used as a positive control. After irradiation of the targeted molecule, 10 μM DCFH-DA was added to the suspension and incubated for 30 min at 37 °C. Immediately, the DCF fluorescence intensity was measured using a spectrofluorophotometer (JASCO FP-6500, light source Xenon arc lamp, Japan). Generally, the reaction of the non-fluorescent 2,7-dichlorodihydrofluorescein (DCFH) was carried out using different ROS, such as H_2_O_2_, OH˙, and O_2_˙^−^, and also using the reactive nitrogen species (RNS) ˙NO and ONOO- to release 2′,7′-dichlorofluorescein (DCF), a highly fluorescent product.^31^

#### 2.6.1. Determination of ROS-type releases inside the fungal cells

##### 2.6.1.1. Determination of H_2_O_2_ radicals

In order to determine the most potent ROS radical producer, the quantitative assay method for the H_2_O_2_ determination was conducted after irradiation in a 96-well microplate in duplicate as described in ref. 32. A spectrophotometric microplate assay based on a horseradish peroxidase (HRP)-coupled reaction using *o*-phenylenediamine (OPD) as an H_2_O_2_ probe was carried out. Briefly, 10 μL of the crude fungal extract was incubated using 90 μL of the reaction mixture containing 0.81 U mL^−1^ of HRP and 2 mM of OPD (as a substrate for peroxidase) in 50 mM of Tris–HCl, pH 8.0 buffer at 37 °C. After 1 h, the reaction was terminated by adding 20 μL of 2 M H_2_SO_4_. The amount of the product (H_2_O_2_) was estimated by measuring the absorbency at 490 nm and the specific H_2_O_2_ activity was determined using a calibration curve of known concentrations of freshly prepared H_2_O_2_.

##### 2.6.1.2. Analysis of generated singlet oxygen *via* 1,3-diphenylisobenzofurane (DPIBF) oxidation

Singlet oxygen (^1^O_2_) is one of the highly-reactive oxygen species that play a vital role in antibacterial and antifungal eradication mechanisms. To estimate the ^1^O_2_, the relationship between the concentration of the targeted samples and changes in the absorption peak of DPIBF at 420 nm was analyzed in the presence of the tested fungal strain under optimized irradiation conditions. The DPIBF was initially dissolved in absolute ethanol (99.5%) to prepare a stock solution with a final concentration of 4 mM. In this regard, 15 μL of the DPIBF solution and 150 μL of the tested compound were combined in a cuvette to obtain a final concentration of 1 mM of DPIBF and different concentrations of the targeted compounds (0, 1, 5, 10, 20, and 40 μM). After incorporating fungal-tested strains, the samples were irradiated under the optimized laser conditions. Then, immediately, each of the samples was centrifuged at 8000 rpm for 15 min and the absorption peak of DPIBF was analyzed using a spectrophotometer (Agilent Cary-100, Germany). In addition, the relationship between the changes in the absorption peak of DPIBF and irradiation time were investigated for different exposure times (10, 15, and 20 min) at 420 nm in comparison to the standard photosensitizer compound Rose Bengal according to ref. 33. The changes in the DPIBF absorption peak were reflected in the generation of ^1^O_2_ from the targeted compounds.

### 2.7. Data analysis

The results in this study are reported as the mean ± S.D.

## 3. Results and discussion

### 3.1. Chemistry

**Scheme 1:**
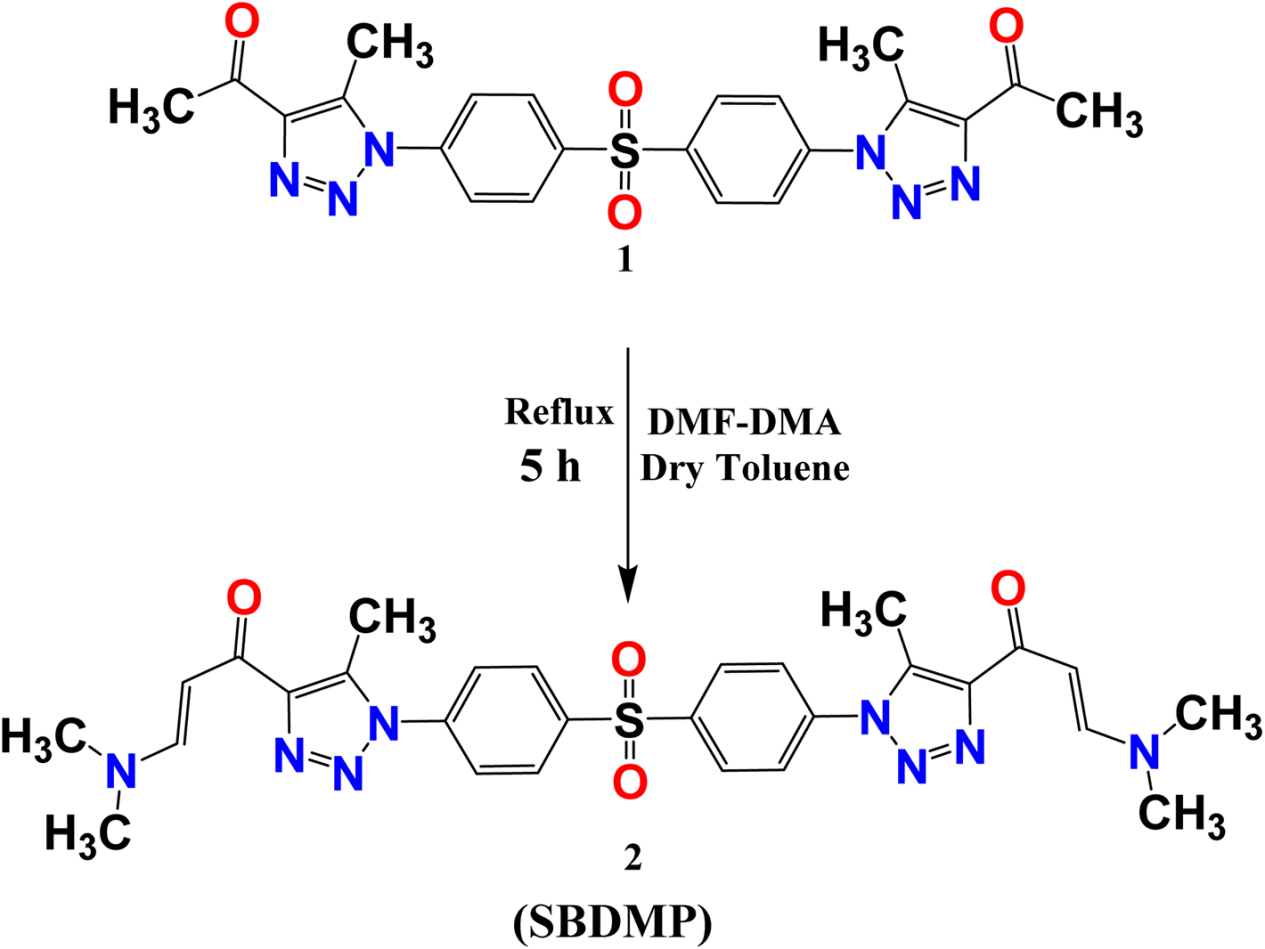
Synthesis of 1,1′-((sulfonylbis(4,1-phenylene))bis(5-methyl-1*H*-1,2,3-triazole-1,4-diyl))bis(3-(dimethylamino)prop-2-en-1-one) (SBDMP) (2).

**Fig. 2:**
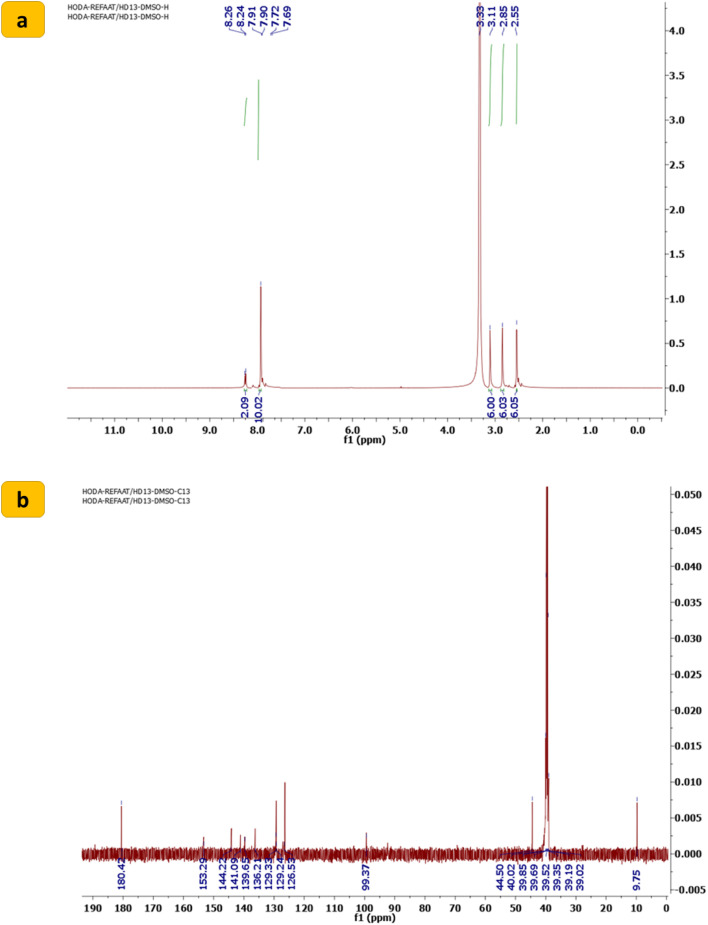
(a) ^1^H NMR spectrum and (b) ^13^C NMR spectrum of 1,1′-((sulfonylbis(4,1-phenylene))bis(5-methyl-1*H*-1,2,3-triazole-1,4-diyl))bis(3-(dimethylamino)prop-2-en-1-one) (SBDMP) (2).

Refining linker conjugation between the 1,2,3-triazoles, sulfone, and other bioactive derivatives is expected to boost their potential activities. To achieve this, 1,1′-((sulfonylbis(4,1-phenylene))bis(5-methyl-1*H*-1,2,3-triazole-1,4-diyl))bis(ethan-1-one) (1) was reacted with DMF-DMA to afford the desired enaminone derivative SBDMP (2) (Scheme 1). The chemical structure of the newly synthesized enaminone derivative SBDMP (2) was affirmed by assigning its spectral and microanalytical data, in which its ^1^H NMR spectrum exhibited three singlet signals at 2.55, 2.85, 3.11 ppm for the protons of six methyl groups. Additionally, the aromatic protons along with the (CH=CH) protons produced a multiplet signal in the region from *δ* 7.69 to 7.91 in addition to a doublet signal at 8.26 ppm (Fig. 2a). Meanwhile, the ^13^C NMR spectrum showed characteristic signals at *δ* 9.75 for (2CH_3_), 44.50 for (4 CH_3_), 99.37 for (2CH=), 126.53 for (2 Ar), 129.24 (4 Ar), 136.21 for (4 Ar), 139.65 for (2 Ar), 141.09 for (2 Ar), 144.22 for (2 Ar), 153.29 for (2N–CH=) and at *δ* 180.42 assigned for (2C=O) (Fig. 2b).

### 3.2. Characterization of SBDMP-loaded silica nanoemulsion

#### 3.2.1. Particle shape and average size characterization

**Fig. 3:**
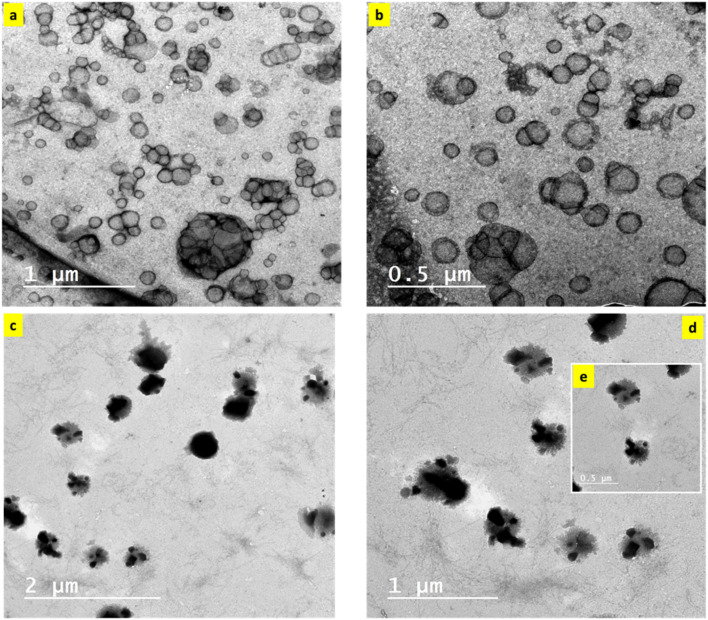
TEM of nanoemulsions (a, b) without SBDMP and (c–e) with SBDMP loading.

The size and surface morphology of silica (Fig. 3a, b) and SBDMP-loaded silica (Fig. 3c–e) nanoemulsions were further examined using TEM. The images show that the emulsion droplets are spherical in shape, with a small size. As observed from the TEM images (Fig. 3a and b), silica nanoemulsion samples were assessed at different magnifications to clarify the nature of these particles. Spherical particles with cavities were observed for the silica nanoemulsion. These cavities are available to be encapsulated with any model drugs or other organic compounds such as SBDMP. Moreover, by examining the feature of silica nanoemulsion loaded with SBDMP (Fig. 3c–e), the particles were found to be black in color and with no cavities signifying that the SBDMP was successfully encapsulated inside the cavity of silica nanoparticles.

**Fig. 4:**
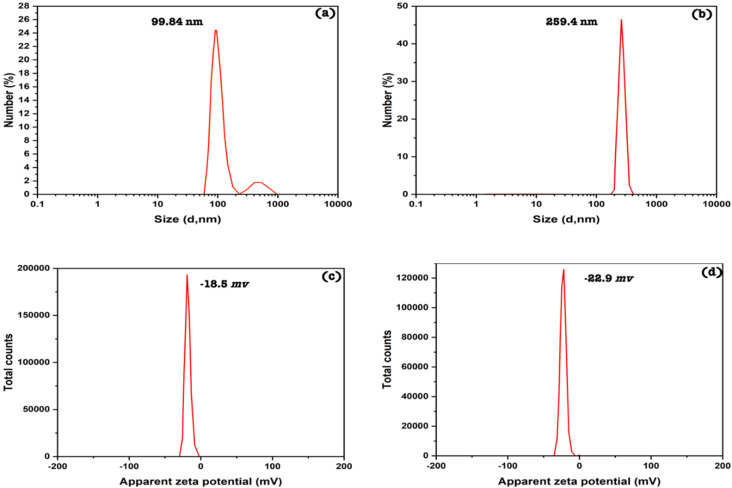
Particle sizes of the nanoemulsions (a) without SBDMP and (b) with SBDMP, zeta potentials of the nanoemulsions (c) without SBDMP and (d) with SBDMP loading.

The average diameter of silica nanoemulsion with and without SBDMP loading was further evaluated using DLS (Fig. 4a and b). Taking into consideration, the obtained values are the average of many runs or cycles of measurments. It was observed that nanoparticles exhibited an average size around 100 nm (99.84 nm) (Fig. 4a). The size of silica nanoemulsion was increased to 259 nm (Fig. 4b) when loaded with SBDMP. The increment in the average size can be attributed to the adsorption of SBDMP onto the outer surface of silica nanoemulsion, which led to enlargement the size of the formed these particles.

#### 3.2.2. Zeta potential characterization of silica nanoemulsion with and without SBDMP loading

The zeta potential of silica nanoemulsion and SBDMP-loaded silica nanoemulsion was measured to predict the stability and to evaluate the effect of SBDMP loading (Fig. 4c and d). As shown in Fig. 4c, silica nanoemulsion exhibited surface charge (zeta potential) with a value equal to −18.5 mV. Meanwhile, the value of SBDMP-loaded silica nanoemulsion became −22 mV. It was observed that the difference in the two values of both silica nanoemulsion and SBDMP-loaded silica nanoemulsion is very slight. Generally, zeta potential values that exceed 30 mV (positive or negative) are ideal for a stable colloidal system.

However, the zeta value close to −30 mV in this study indicates that the nanoemulsion is sterically stable due to the effect of Pluronic F-68 which prevent the agglomeration of nanoparticles. It was also observed that there was no sign of phase separation or separation for both samples (silica nanoemulsion and SBDMP-loaded silica nanoemulsion).

### 3.3. The optical properties of the SBDMP and SBDMP-loaded silica nanoemulsion

#### 3.3.1. UV-vis-NIR optical absorption characterization

The study of the optical properties of the SBDMP and SBDMP-loaded silica nanoemulsion is one of the most important studies by which to identify if the mechanism of their antifungal treatment by laser-induced photodynamic therapy stems from phototoxicity or the production of ROS inside the cell.

**Fig. 5:**
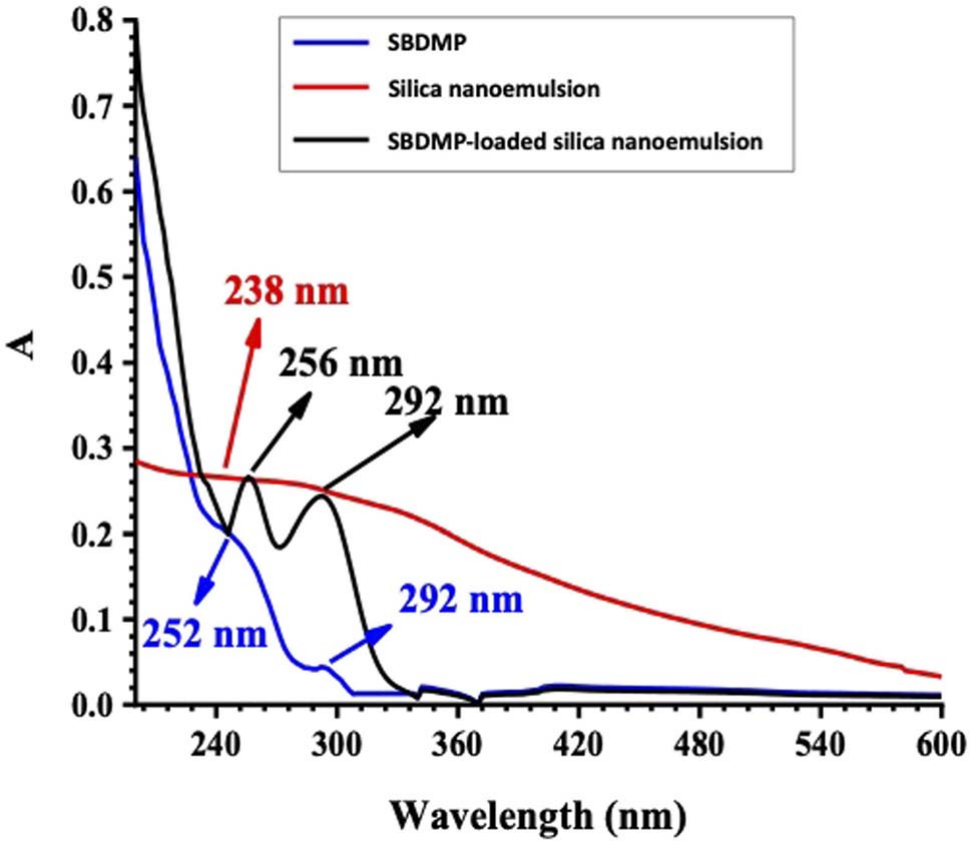
UV optical absorption spectra of the SBDMP, silica nanoemulsion, and SBDMP-loaded silica nanoemulsion.

The UV optical absorption spectra of SBDMP, silica nanoemulsion, and SBDMP-loaded silica nanoemulsion with concentrations of 10 μg mL^−1^ suspended in distilled water are presented in Fig. 5. The UV spectra of the SBDMP show only the two most significant UV bands of the dapsone and its derivatives with some wavelength shifts.^34–36^ The first absorption band has a peak at 252 nm corresponding to an electronic transition from the electronic ground state to the sixth electronic excited state, attributed to the HOMO−1 of the nitrogen lone pair — pi (π_LP-N_) to the LUMO of the sigma antibonding orbital of the sulfur–oxygen transition (σ*_SO_). The second absorption band at 292 nm corresponds to the one-electron HOMO (π_LP-N_) → LUMO (σ*_SO_) transition. This result is in agreement with the results of Moura *et al.*^34^

The UV-vis absorption spectra of silica nanoemulsion with a particle size of 100 nm exhibit a broad absorption band at 238 nm corresponding to paramagnetic defects such as paramagnetic positively-charged oxygen vacancies (≡Si·Si≡) and neutral dangling Si bonds (≡Si).^37–40^ The absorption spectra of SBDMP-loaded silica nanoemulsion shows two UV absorption peaks of SBDMP sharpened with high absorption intensity. This shows that SBDMP was successfully loaded and encapsulated inside the cavity of silica nanoemulsion.^7^ The increase in the absorption intensity according to the Lambert–Beer law refers to an increase in the concentration of the SBDMP due to the some of the SBDMP being adsorbed onto the outer surface of the silica nanoemulsion. The first peak of the SBDMP is located at 256 nm with a 4 nm blue wavelength shift while the second peak is still at 292 nm due to the increase in the size of the silica nanoemulsion particles from 100 nm to 259 nm upon loading of the SBDMP.^29,41^ These results are in agreement with TEM results and zeta potential evaluations. The SBDMP absorption spectra shows that any photo-induced activity by the developed silica nanoemulsion is initiated by two-photon absorption (TPA). TPA is based on substituting the one-photon absorption (OPA) from the ground state to an excited state in one step *via* two absorption steps concurrently throughout the lifetime of the first excited state.^42,43^ In addition to its limited destroying effects, perfect regional selectivity, enhanced LIPDT, and deep penetration into biological tissues, TPA has become an effective strategy in life sciences.^42–45^

#### 3.3.2. PL characterization

**Fig. 6:**
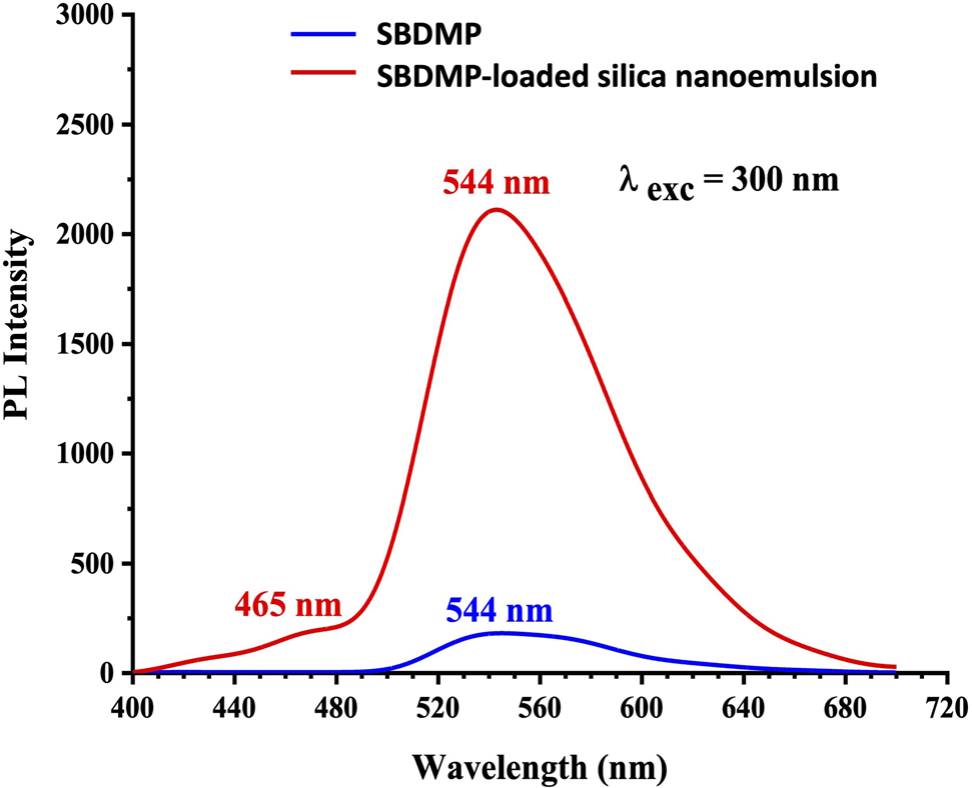
Photoluminescence spectra of the SBDMP and SBDMP-loaded silica nanoemulsions.

The PL emission spectra of the 10 μg mL^−1^SBDMP and SBDMP-loaded silica nanoemulsion samples suspended in distilled water are shown in Fig. 6. The PL emission spectrum of the SBDMP excited by 300 nm shows only one emission peak centered at 544 nm with low relative emission intensity. This one dominant emission peak can be attributed to the electron transition from the HOMO (π_LP-N_) → LUMO (σ*_SO_) of dapsone, while SBDMP-loaded silica nanoemulsion shows two emission peaks, where the one at 544 nm is similar to the emission peak of SBDMP with a fourth multiplicative relative emission intensity. This can be attributed to an increase in the absorption coefficient of SBDMP by silica nanoemulsion, as shown in Fig. 5.^7,29,46^ The second small emission band at 465 nm can be attributed to the emission of silica nanoemulsion that is deposited on the surface of SBDMP-loaded silica nanoemulsion. This emission band is induced by electron–hole recombination between self-trapped excitons and oxygen-deficient centers in silica.^40,47–49^

Based on the results of the optical absorption and photoluminescence emission spectra of SBDMP and SBDMP-loaded silica nanoemulsion, it was found that, the tested compounds do not absorb the 645 nm wavelength light used in the study of the antifungals *via* laser-induced photodynamic therapy. The PL emission spectra of SBDMP also show photo-induced activity by silica nanoemulsion initiated by TPA as in the UV-vis absorption spectra. The studied optical properties verified that, SBDMP-loaded silica nanoemulsion exhibits very deep penetration into biological tissues due to the TAP mechanism.^42,43^ This directs the study to the fact that the liberation of ROS inside the cell is considered to be the dominant influence in this phenomenon, as it will be studied in detail in the following.

### 3.4. Anti-mucormycosis activity of the tested molecules

Opportunistic fungi such as Mucorales strains still exhibit a significant medical hindrance due to their high infection rates among immunocompromised patients as a result of their rapid proliferation and remarkable resistance to classical broad-spectrum antibiotics. Therefore, discovering and developing a new therapeutic route to overcome these factors has become an urgent task. Among the advanced therapeutic strategies, irradiation by LIPDT to induce the generation of ROS resulting from excited molecules penetrating the complicated fungal cell wall easily allowed the target molecules to enter inside the cells, which in turn led to fast disruption of the fungal organelles. Herein, the anti-mucormycosis activities of the targeted molecules were preliminary investigated according to the standard agar well diffusion procedure in comparison to the antifungal agents fluconazole and amphotericin B.

**Fig. 7:**
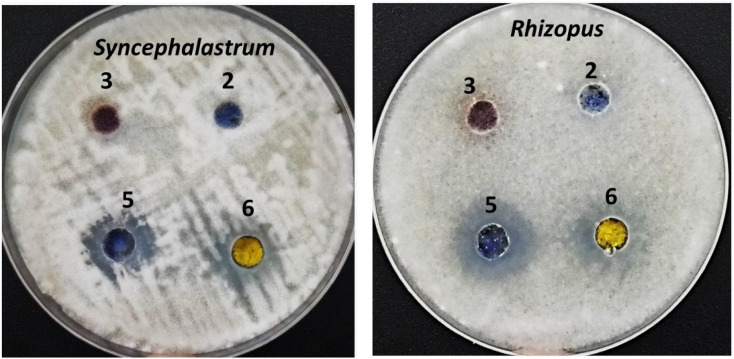
Anti-mucormycosis activity of the synthesized compounds.

As shown in Fig. 7, the compounds corresponding to SBDMP, (5), and SBDMP-loaded silica nanoemulsion (6) showed better inhibition activity against *Rhizopus microsporous* and *Syncephalastrum racemosum* than that observed for the antifungal agent. In addition, weak anti-mucormycosis activity was observed for silica nanoemulsion (3). The tested Mucorales strains proved to be more resistant to fluconazole even at 100 μg mL^−1^, however the proliferation of tested strains was affected by amphotericin B at 62.5 μg mL^−1^ which emphasized the potency of the selected molecules. Furthermore, based on the CFU procedure, the inhibition percentage of SBDMP-loaded silica nanoemulsion occurred with 14.6 ± 1.5% for *Rhizopus microsporous* and with 22.2 ± 0.66% for *Syncephalastrum racemosum*; however, SBDMP showed inhibition activity of 9.3 ± 0.02% and 16.8 ± 0.25% toward *Rhizopus microsporous* and *Syncephalastrum racemosum*, respectively (Table 1).

**Table 1:** Inhibition activity of the synthesized compounds against *Rhizopus microsporous* and *Syncephalastrum racemosum*

Sample	Inhibition in Mucorales survival, CFU (%)	
	*Rhizopus microsporous*	*Syncephalastrum racemosum*
Dapson	3.29 ± 0.11	2.15 ± 0.20
SBDMP	9.3 ± 0.02	16.8 ± 0.25
Silica nanoemulsion	1.76 ± 1.7	3.16 ± 0.91
SBDMP-loaded silica nanoemulsion	14.63 ± 1.5	22.2 ± 0.66
Fluconazole	4.73 ± 0.75	5.11 ± 0.5
Amphotericin B	22.73 ± 1.77	29.53 ± 1.5
Control	100	

Moreover, SBDMP and SBDMP-loaded silica nanoemulsion were subjected to study under irradiation conditions to evaluate their photo-killing activity against Mucorales strains. Firstly, screening of the targeted molecules under two laser wavelengths, 515 nm (blue light) and 645 nm (red light), was conducted at 50 mW for 15 min, as shown in the results in Table 2, of which the most potent eradication of fungal cells was obtained when irradiation was conducted under red laser light (645 nm). Furthermore, the most efficient response to the irradiation parameters was achieved by the SBDMP-loaded silica nanoemulsion, where its photoinactivation ability against *Rhizopus microsporous* and *Syncephalastrum racemosum* reached 19.4 ± 0.02% and 36.8 ± 0.52%, respectively. Meanwhile, the irradiation response of SBDMP showed moderate activity against *Syncephalastrum racemosum* (24.8 ± 0.42%) and slight activity against *Rhizopus microsporous* (14.8% ± 0.32) when compared to treatment without irradiation. In addition, the exposure of the tested organisms directly toward laser wavelengths only (without targeted molecules) was found to be correlated with a lower inhibition activity, even after 30 min (Table 2), in which the inhibition activity did not exceed 10%.

**Table 2:** Response of targeted molecules toward the wavelengths 515 and 638 nm

Sample	Inhibition in Mucorales survival, CFU (%)							
	*Rhizopus microsporous*				*Syncephalastrum racemosum*			
	Dapsone	SBDMP	Silica nanoemulsion	SBDMP-loaded silica nanoemulsion	Dapsone	SBDMP	Silica nanoemulsion	SBDMP-loaded silica nanoemulsion
Without laser irradiation	3.29 ± 0.11	9.3 ± 0.02	1.76 ± 1.7	14.63 ± 1.5	2.15 ± 0.20	16.8 ± 0.25	3.16 ± 0.91	22.2 ± 0.66
Under irradiation at 515 nm	7.3 ± 0.12	12.3 ± 0.32	9.6 ± 0.5	16.8 ± 0.22	16.8 ± 08	21.3 ± 0.22	11.8 ± 0.12	22.8 ± 0.35
Under irradiation at 645 nm	8.76 ± 1.7	14.8 ± 0.32	9.3 ± 0.12	19.4 ± 0.02	3.16 ± 0.91	24.8 ± 0.42	13.3 ± 0.15	36.8 ± 0.52
Treated with laser only at 645 nm	6.92 ± 0.26				9.26 ± 0.44			
Treated with laser only at 515 nm	7.77 ± 1.55				6.39 ± 0.9			
Fluconazole	4.73 ± 0.75				5.11 ± 0.5			
Amphotericin B	22.73 ± 1.77				29.53 ± 1.5			
Control	100							

**Fig. 8:**
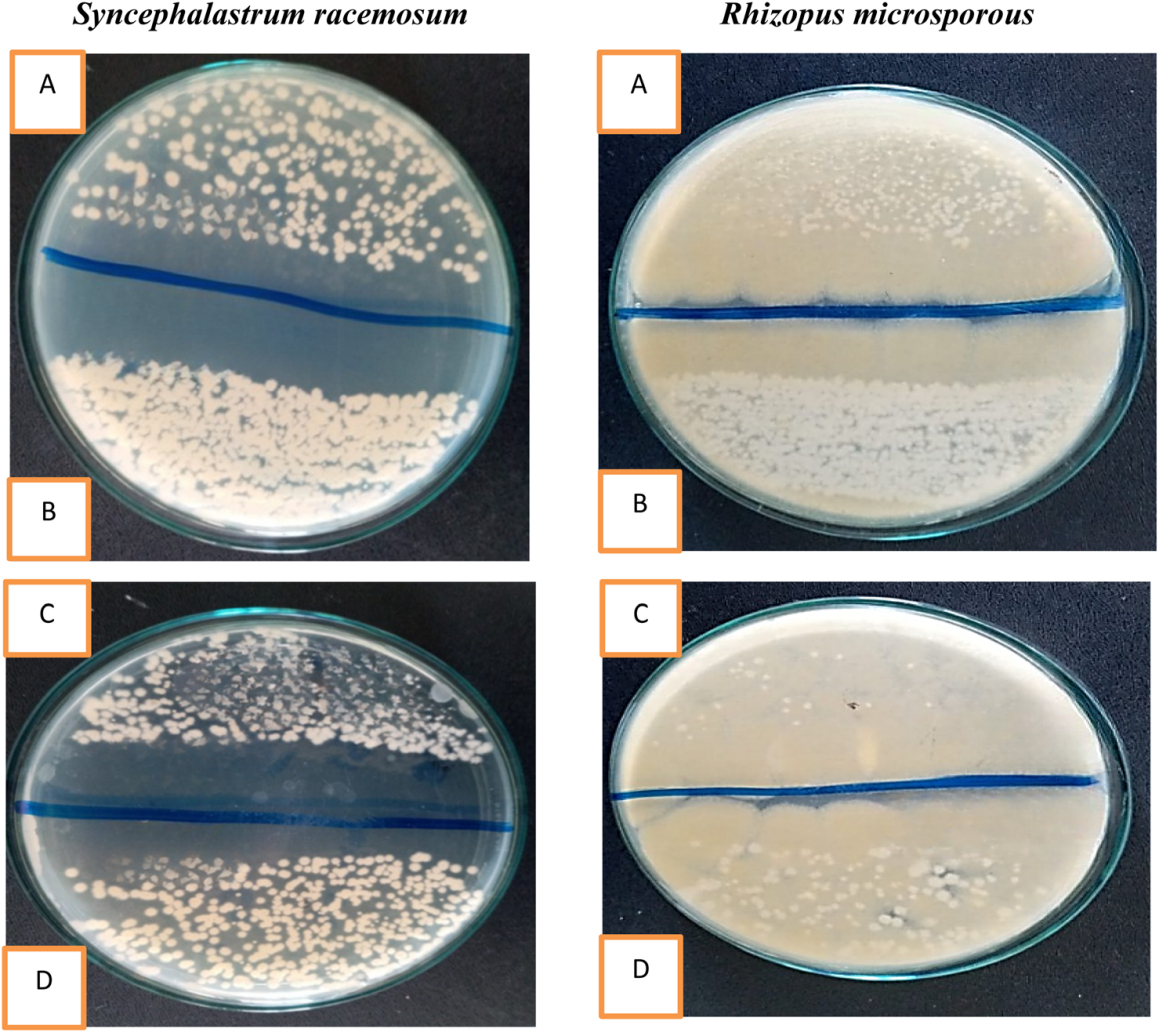
Effect of different irradiation parameters, (A) irradiation SBDMP-loaded silica nanoemulsion by 645 nm, (B) irradiation SBDMP-loaded silica nanoemulsion by 515 nm, (C) irradiation SBDMP-loaded silica nanoemulsion by 645 nm at 184 mW, and (D) irradiation SBDMP-loaded silica nanoemulsion by 645 nm at 50 mW in the vegetative cells of fungal strains.

Secondly, different powers of red light were investigated for 15 min (*i.e.*, 50 mW, and 184 mW). As can be seen in Table 3, the increase of power activity was directly proportional to the increase in the photoinactivation ability, where for the SBDMP-loaded silica nanoemulsion, the eradication of *Rhizopus microsporous* and *Syncephalastrum racemosum* at 184 mW was carried out with results of 30.3 ± 2.8% and 47.5 ± 3.52%, respectively (Fig. 8). Moreover, the photokilling activity of SBDMP also increased along with the increase in the power activity against *Syncephalastrum racemosum* (34.9 ± 2.22% at 184 mW); however, the increase in the eradication activity was found to be low against *Rhizopus microsporous* even at 184 mW (24.1 ± 0.52%). Examination of the photoinactivation against the tested strains under different powers without the target molecules also showed lower activity, not exceeding 15% for *Rhizopus microsporous* and 10% for *Syncephalastrum racemosum*.

**Table 3:** Response of the target molecules toward 638 nm at 50 mW and 184 mW

Sample	Inhibition in Mucorales survival, CFU (%)							
	*Rhizopus microsporous*				*Syncephalastrum racemosum*			
	Dapsone	SBDMP	Silica nanoemulsion	SBDMP-loaded silica nanoemulsion	Dapsone	SBDMP	Silica nanoemulsion	SBDMP-loaded silica nanoemulsion
Without laser irradiation	3.29 ± 0.11	9.3 ± 0.02	1.76 ± 1.7	14.63 ± 1.5	2.15 ± 0.20	16.8 ± 0.25	3.16 ± 0.91	22.2 ± 0.66
Under power at 50 mW	15.76 ± 1.7	13.9 ± 0.32	9.3 ± 0.12	19.4 ± 0.02	3.16 ± 0.91	27.9 ± 0.42	13.3 ± 0.15	36.8 ± 0.52
Under power at 184 mW	19.76 ± 1.7	24.1 ± 0.52	16.3 ± 0.4	30.3 ± 2.8	11.6 ± 0.11	34.9 ± 2.22	17.3 ± 0.15	47.5 ± 3.52
Laser only at 645 nm, 50 mW	6.92 ± 0.26				9.26 ± 0.44			
Laser only at 645 nm, 184 mW	13.89 ± 1.05				9.45 ± 0.22			
Fluconazole	4.73 ± 0.75				5.11 ± 0.5			
Amphotericin B	22.73 ± 1.77				29.53 ± 1.5			
Control	100							

Thirdly, the determination of the optimal time for significant photoinactivation was also carried out from 5–30 min. In this regard, high eradication activity was achieved after irradiation for 20 min and after that the inhibition activity was relatively constant (data not shown). Subsequently, the preparation of different concentrations of SBDMP-loaded silica nanoemulsion and SBDMP was conducted in order to evaluate the minimum inhibition concentration (MIC) under the optimized irradiation conditions as determined previously. Each of the compounds was prepared at concentrations from 5 to 400 μL and then incubated with the tested fungal strain for 20 min in the dark. After that, each concentration was exposed to laser light under optimal conditions.

The effect of fungal growth in the presence of the optimized conditions was observed, as presented in Fig. 8, as the optimal conditions that led to the maximum inhibition ratio were observed under irradiation at 645 nm at a power of 50 mW in the presence of the SBDMP-loaded silica nanoemulsion.

As shown in Table 4, the superiority of SBDMP-loaded silica nanoemulsion at lower concentration was indicated, at which the maximum eradication activity was established at 62.5 ± 6.42 μg mL^−1^ and 31.125 ± 2.42 μg mL^−1^ against *Rhizopus microsporous* and *Syncephalastrum racemosum*, respectively. Noticeably, enhancement of eradication activity under the optimized irradiated conditions was demonstrated to be quite high at five-fold for *Rhizopus microsporous* and eight-fold for *Syncephalastrum racemosum* when compared to the molecules without irradiation. Meanwhile, the MIC value of the SBDMP was observed to be 100.5 ± 4.31 μg mL^−1^ against *Rhizopus microsporous* and 125 ± 1.5 μg mL^−1^ toward *Syncephalastrum racemosum*. Importantly, the greatest MIC value of the irradiated SBDMP and SBDMP-loaded silica nanoemulsion for each Mucorales strain was obviously higher than that obtained for the standard antifungal agents.

**Table 4:** Minimum inhibitory concentration (MIC) of the selected compounds under the optimized irradiation conditions

Sample no.	Minimum inhibitory concentration (MIC, μg mL^−1^)							
	*Rhizopus microsporous*				*Syncephalastrum racemosum*			
	Dapsone	SBDMP	Silica nanoemulsion	SBDMP-loaded silica nanoemulsion	Dapsone	SBDMP	Silica nanoemulsion	SBDMP-loaded silica nanoemulsion
Without laser irradiation	<400	***350*** *± 6.42*	<400	300 ± 5.22	<400	200 ± 5.75	<400	250 ± 1.55
Under optimized irradiation condition	300 ± 7.22	100.5 ± 4.31	<400	62.5 ± 6.42	160 ± 5.52	125 ± 1.5	<400	31.125 ± 2.42
Fluconazole	<400				<400			
Amphotericin B	125 ± 2.55				62.5 ± 1.75			

The Mucorales species is a common pathogenic fungus in humans, and owing to the restricted available antifungal agents, the development of new advanced drugs is an urgent issue. Development of anti-Mucorales strategies using advanced techniques could open up a promising way to achieve the rapid elimination of mucormycosis diseases, due to their fast virulence and resistance to classical antifungal agents. The photosensitive response of newly synthesized composites based on organic compounds and nanomaterials was exploited in this report to study their potentiality in the eradication of Mucorales strains. Thus, we focused on the preparation of organic compounds based on dapsone and triazole molecules and loaded the composites onto silica nanoemulsion, and investigated them under irradiation toward Mucorales pathogens, which showed significant eradication of them in the red-light region. In this way, the selection of dapsone was due to it being a common sulfone compound that exhibits broad-spectrum antibiotic properties and photo-response toward different wavelengths of light. Dapsone is a sulfone antibiotic and anti-inflammatory agent that has been implicated in both phototoxic and photoallergic drug eruptions.^50,51^ This has been confirmed both by oral drug rechallenge and photo patch testing.^51,52^ In addition, the incorporation of triazole molecules with dapsone has also been implicated in photosensitivity toward laser light, which could be more active under irradiation conditions.^53^

### 3.5. ROS generation

**Fig. 9:**
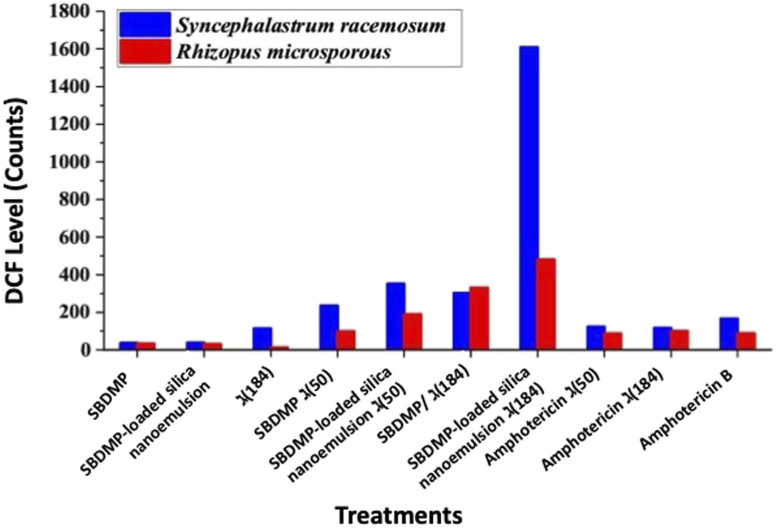
ROS quantification inside the fungal cells after treatment.

The predicted mechanism of action for the irradiation treatment in the presence of the targeted compounds is ROS generation. Therefore, quantification of the ROS inside the damaged fungal cells was examined using fluorescent DCFH. DCFH was rapidly oxidized by the intracellular ROS and converted into the highly fluorescent molecule, DCF.^8^ Accordingly, the proliferation of the tested fungal strains under irradiation of laser light alone was correlated with a lower ROS generation (Fig. 9). Likewise, the treatment of the Mucorales strains with SBDMP and SBDMP-loaded silica nanoemulsion without laser irradiation showed a small amount of ROS release, which was comparable with previous inactivation results. In addition, the moderate ROS generation inside the fungal cells under 50 mW power in the presence of each of the targeted compounds was also observed. The highest amount of ROS was observed under the optimal conditions of red light at 184 mW in the presence of the SBDMP-loaded silica nanoemulsion, particularly against *Syncephalastrum racemosum*, which exhibited notable fluorescence increases. Overall, efficient ROS production was obtained for the irradiated SBDMP-loaded silica nanoemulsion than that produced for the irradiated SBDMP against both fungal strains. Furthermore, the antifungal agent amphotericin B was also irradiated under the same condition; there was no enhancement in the ROS generation when it was used alone. In comparison, the efficacy of the irradiated SBDMP-loaded silica nanoemulsion to the release of ROS proved to be more distinguishable than amphotericin B. In fact, the intracellular production of ROS is a major and well-known response to antifungal agents in fungal cells. Amphotericin B is one of the ROS induced in a mitochondria-dependent manner in several pathogenic fungal pathogens such as *Candida albicans*, *Cryptococcus neoformans*, and *Aspergillus fumigatus*. In accordance with our results, an anti-mucormycosis compound, SBDMP-loaded silica nanoemulsion, increases the induction of the ROS level, which suggests that oxidative stress intracellularly causes growth inhibition.^54^ Observably, one of the compounds tested, SBDMP, triggered ROS production in the hyphae of the Mucorales strains under laser light exposure. To our knowledge, this is the first study of the significant anti-mucormycosis activity of SBDMP-loaded silica nanoemulsion under PDA. It has been reported that the antimicrobial activity of dapsone inhibits the synthesis of dihydrofolic acid by competing with *para*-aminobenzoic acid for the active site of dihydropteroate synthetase, in which it was examined against various pathogenic bacteria such as *streptococci*, *staphylococci*, *pneumococci*, *mycobacteria*, and other strains.^17^

#### 3.5.1. Determination of ROS type

**Fig. 10:**
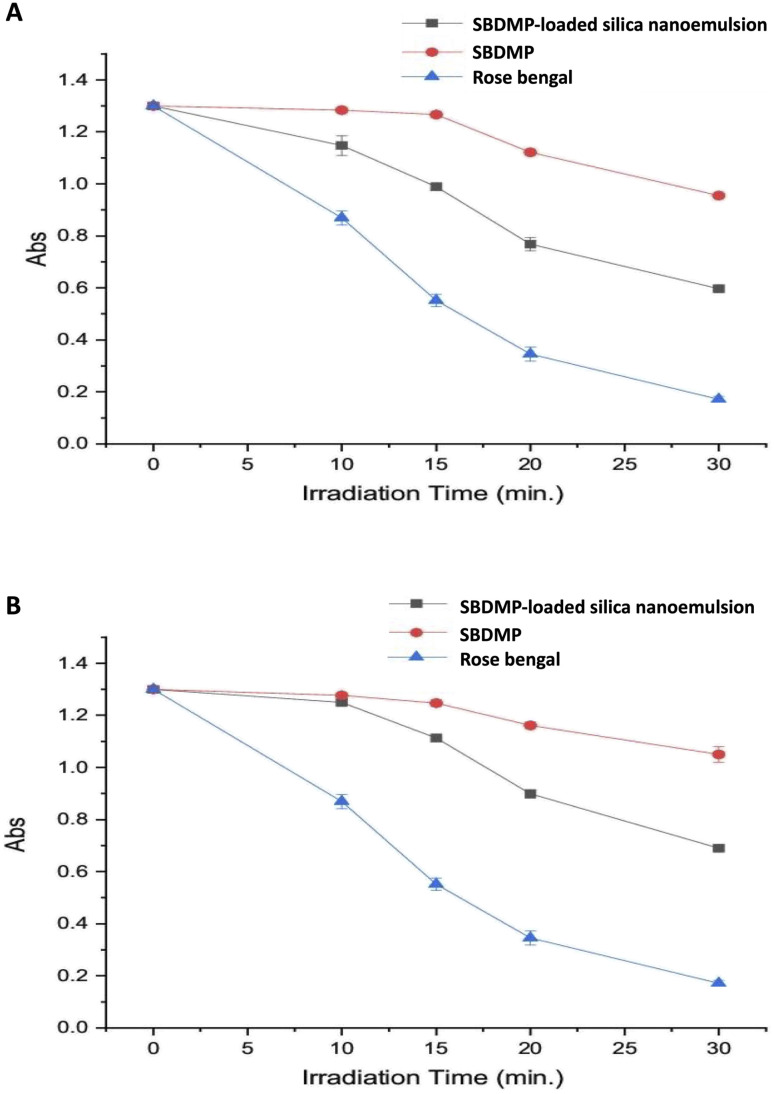
Quenching of DPIBF (1 mM) at 420 nm. The absorbance (Abs) decreased with an increase in the irradiation time compared to the standard photosensitizer rose bengal (RB) over 30 min. DPIBF was oxidized by singlet oxygen photogenerated against (A) *Syncephalastrum racemosum* and (B) *Rhizopus microsporous*.

After quantification of the intracellular ROS, the determination of the ROS generated type was also interesting in order to clearly demonstrate the specific type that is released from the targeted SBDMP and SBDMP-loaded silica nanoemulsion under irradiation. H_2_O_2_ and ^1^O_2_ were investigated under optimized irradiation conditions, in which H_2_O_2_ radicals were observed at a low concentration for both targeted compounds even after 20 min of irradiation (data not shown). Meanwhile, the detection of ^1^O_2_ was remarkably increased with an increase in exposure time (Fig. 10), where the highest quenching of the DPIBF at 420 nm was obtained after 30 min for the SBDMP-loaded silica nanoemulsion against both fungal pathogens.

The most ^1^O_2_ release commonly occurs from a photosensitizer molecule that is excited by laser light with different wavelengths *via* energy transfer to triplet oxygen. The generated ^1^O_2_ is mainly responsible for high cytotoxicity in the LIPDT.^55^ Our findings revealed a low amount of H_2_O_2_ release, suggesting that the generated ROS, such as H_2_O_2_, is rapidly converted into ^1^O_2_ through the catalytic actions of redox-active metals. Otherwise, through ground state reactions, photoinduced processes of different molecules can release ^1^O_2_, in which the ^1^O_2_ could be generated from triplet oxygen based on photoirradiation at a wavelength nearing 600 nm.^56^

## 4. Conclusion

It is clear that silica nanoemulsion and SBDMP-loaded silica nanoemulsion were synthesized using a high-intensity ultrasonic method in the presence of Pluronic F-68 as an efficient surfactant. Silica nanoemulsion can be efficiently functionalized with SBDMP thanks to ultrasonic-assisted synthesis. Silica nanoemulsion and SBDMP-loaded silica nanoemulsion were successfully fabricated with a small size and good distribution, as depicted from TEM, DLS, and zeta potential measurements. The silica nanoemulsion loaded with SBDMP exhibited a size of around 260 nm and a zeta potential of around −22.9 mV. The utilization of SBDMP-loaded silica nanoemulsion under LIPDT proved to be efficient in preventing the proliferation of Mucorales strains. In addition, it exhibited deep penetration into biological tissues due to a two absorption photon (TAP) mechanism. The release of ROS from the irradiated SBDMP-loaded silica nanoemulsion was better than that of amphotericin B, making it a promising candidate for the treatment of mucormycosis disease.

## Conflicts of interest

There are no conflicts to declare.

## Supplementary Material
